# Cognitive Bias, Entrepreneurial Emotion, and Entrepreneurship Intention

**DOI:** 10.3389/fpsyg.2020.00625

**Published:** 2020-04-29

**Authors:** Yijun Zhao, Baoguo Xie

**Affiliations:** ^1^Economics and Management School, Wuhan University, Wuhan, China; ^2^School of Management, Wuhan University of Technology, Wuhan, China

**Keywords:** cognitive bias, entrepreneurial emotion, entrepreneurship intention, optimism, overconfidence

## Abstract

Although numerous studies have explored the factors influencing entrepreneurial activity, there is a lack of a theoretical basis for linking these factors to entrepreneurship behavioral intention. The current study uses the theory of self-regulating attitude to construct a theoretical model of examining the relationship among cognitive bias, entrepreneurial emotion, and entrepreneurship intention. A total of 312 valid samples were collected from college students at a Chinese university. The bootstrapping method was used to test the multi-mediation hypotheses. Our research found that positive entrepreneurial emotion plays a mediating role in the relationship between optimism and entrepreneurship intention, whereas negative entrepreneurial emotion plays a mediating role in the relationship between overconfidence and entrepreneurship intention. These findings underline the importance of a correct understanding of cognitive bias and entrepreneurial emotion in the process of entrepreneurship.

## Introduction

Entrepreneurship plays a vital role in economic development, job creation, and social welfare (Poblete et al., 2019; Ravenelle, 2019). However, entrepreneurial behaviors are not growing as quickly as expected (Randerson et al., 2020). In particular, individuals with similar demographic characteristics have large differences in their entrepreneurial behaviors (Obschonka et al., 2012; Amarakoon et al., 2019). What causes these individuals to differ in their intention to start a business?

Entrepreneurial traits and entrepreneurship cognition theory explain the impact of personality traits, achievement needs, control focus, risk-taking, and other factors on entrepreneurial behavior from the perspective of individual entrepreneurs (Obschonka et al., 2012; Neneh, 2019; Perez-Lopez et al., 2019). However, entrepreneurial characteristics, which are individually owned cognition and judgment, are distinct (Wang et al., 2019). Thus, there is still space for further study of the differences in entrepreneurial cognition shown by individuals. It is not appropriate to use cognitive mechanisms to predict who will choose to become entrepreneurs without considering the sources of cognition. Perez-Lopez et al. (2019) point out that the core of entrepreneurship cognition theory should focus on cognitive characteristics and how they influence individual attitudes, intentions, and behaviors, emphasizing the important role played by contextual factors in entrepreneurship cognition theory.

In previous studies, the role of entrepreneurial cognitive bias has not attracted enough attention. Cognitive bias is generally considered a negative factor (Krans et al., 2019). Cognitive biases include different types of dimensions, however, such as optimism and overconfidence. With the advantage of quick, minimalist decision-making (Onie et al., 2019), more precise judgments occur due to the lack of resources to reference (Bosmans et al., 2019). In a rapidly changing environment, it is a challenging task for a rational decision-maker to take advantage of all available information and seize opportunities. Once a decision is made, there is no possibility of opportunity. In such a complex environment, different types of cognitive bias will play distinct roles in the creation of entrepreneurial intention (Hahn et al., 2019). Previous literature regarding entrepreneurial intention used the theory of planned behavior (Ajzen, 1991), which considered entrepreneurship a planned behavior of the relevant intention, and defined entrepreneurial intention as mental representations of a person’s propensity to start a business (Obschonka et al., 2015; Gorgievski et al., 2018). Bagozzi (1992) points out that classical attitude theory simplifies the use of general psychological variables to explain social behavior, and simplifies many beliefs and evaluations into an overall, single-dimensional attitude, so these theories lack the explanatory power to change.

Moreover, in previous studies, the important role of entrepreneurial emotion has not attracted sufficient attention, and less systematic research has attempted to explain the potential role of emotion in the entrepreneurial process (Cardon et al., 2012; Hu et al., 2017). Some studies have found, however, that individual decisions at different stages of entrepreneurship are influenced by emotion and reason (Cardon et al., 2012), and significant differences exist in the impact of types of emotions on the assessment of entrepreneurial opportunities (Wolfe and Shepherd, 2015). These studies ignore the role of different types of emotions in the relationship between cognition and entrepreneurship behavior (Doern and Goss, 2013). Indeed, the emotions that an individual or team has on entrepreneurship include both positive and negative emotions (Wolfe and Shepherd, 2015), and different emotional reactions of the individual have distinct effects on the outcomes of behavioral variables.

Bagozzi (1992) points out that behavior is a response activity that stems from an individual’s assessment of the situation and subsequent emotional responses. Specific assessments and desires are functions of unique stimuli that lead to specific emotions and coping responses. The self-regulating process of evaluation, emotional response, and coping response is the core of the theory. Cognitive bias is a characteristic of employees’ perception of the entrepreneurial environment. Individuals generate different emotions and attitudes based on this environmental assessment, which further determine the individual’s entrepreneurial intentions and behavior. Given the preceding arguments, this study follows the theory of self-regulating attitude, establishes a research model of evaluation, emotional reaction, and coping response, and explores the influence of cognitive bias on entrepreneurship intention through the mediating effect of entrepreneur emotions.

## Literature Review and Hypothesis

### Self-Regulating Attitude Theory

On the theoretical basis of traditional attitude, Bagozzi (1992) put forward the theory of self-regulating attitude. The self-regulating process of evaluation, emotional reaction, and coping response is at the heart of the theory (Lazarus, 1991). The theory states that behavior is a response activity that results from an individual’s assessment of the situation and subsequent emotional reaction. Specific assessments and desires are functions of unique stimuli that lead to specific emotions and coping responses. The theory distinguishes between the evaluation process and the emotional reaction process, emphasizing the role of cognitive and emotional self-regulation mechanisms in attitude theory. Thus, it expands the interpretation of social behavior.

**FIGURE 1:**
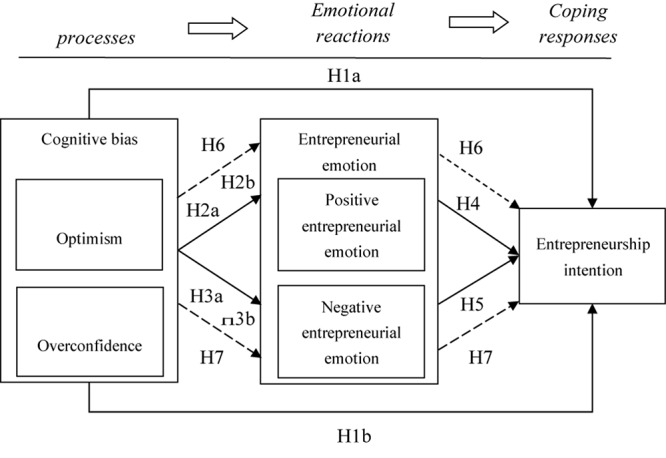
Research model. Dashed lines indicate mediation effects.

Some scholars have applied this theory to the empirical research of employee attitude and behavior (Kruglanski et al., 2015; Rhodes et al., 2016; Hansen and Steinmetz, 2019). However, no scholars have introduced the model into the study of entrepreneurship psychology. Using the theory of self-regulating attitude, this study proposes that cognitive bias is an individual’s assessment of the external working environment and practices. Cognitive bias is considered to be a precursor to an employee’s emotional response. Entrepreneurial emotion is an important emotional response variable in an individual’s entrepreneurship process. Entrepreneurship intention is the behavioral outcome after an individual’s emotional response. The conceptual framework for this study is shown in Figure 1.

### Relationship Between Cognitive Bias and Entrepreneurship Intention

Cognitive bias is an individual’s perceptual deviation from rationality when thinking, reasoning, and making decisions (Alos-Ferrer et al., 2016; Domeier and Sachse, 2016; Marchetti et al., 2019). Different cognitive biases will trigger differences in the perception of the internal and external environment for each individual, which, in turn, will affect their entrepreneurial emotions. Kinari (2016) argued that optimism and overconfidence are closely related to the cognitive bias of entrepreneurship. Optimism refers to the tendency to underestimate the difficulty of task (Heger and Papageorge, 2018), while overconfidence is the tendency to overestimate the chance of positive events (Chaudhary, 2018).

Although cognitive bias is seen as having a negative effect (Giacomin et al., 2016), it may be a cognitive mechanism for making quick decisions (Bernoster et al., 2018). When an entrepreneur is faced with a complex environment, it helps entrepreneurs make quick decisions because cognitive biases do not require much time or cognitive resources. This has led scholars to present consequences of cognitive bias.

In the case of optimism and entrepreneurship intention, optimists ignore uncertainty and are only slightly aware of the level of risk (Trevelyan, 2008). They overrated the chances of successfully starting a real business (Heger and Papageorge, 2018). Optimism also increases entrepreneurs’ commitment to risky causes (Joo and Durri, 2017) and causes delays or helps suspend decisions on unsuccessful schemes (Trevelyan, 2008). Conversely, overconfident entrepreneurs tend to overestimate the probability of a particular outcome, treating assumptions as facts, leading to insufficient searches for information (Zacharakis and Shepherd, 2001). These entrepreneurs fail to gather relevant information, which affects the quality of decision-making, leading to entrepreneurial setbacks, thereby reducing the actual intention to start a business (Hayward et al., 2010). This study posits that the two types of cognitive bias differentially predict entrepreneurship intention:

Hypothesis 1a. Optimism is positively linked with entrepreneurship intention.

Hypothesis 1b. Overconfidence is negatively linked with entrepreneurship intention.

### Relationship Between Cognitive Bias and Entrepreneurial Emotion

Entrepreneurial emotion refers to an emotion held by an individual about entrepreneurship (Cardon et al., 2012). Previous literature on entrepreneurial emotion has not paid much attention to individual cognitive processes. Recent research has indicated that individual decision-making at different stages of entrepreneurship is influenced by emotion and rationality (Grichnik et al., 2011; Doern and Goss, 2013). Additionally, emotion is an important factor influencing employee output and customer service perception behavior and is regulated by situational and organizational factors (Byrne and Shepherd, 2015; Wolfe and Shepherd, 2015; Hu et al., 2017).

Some empirical evidence supports self-regulating attitude theory as a relevant framework for explaining how cognitive bias influences entrepreneurial emotion (Chen et al., 2018). Optimists’ over self-evaluation and overly positive view of future events’ results and plans (Bernoster et al., 2018) easily lead to positive emotions (Giacomin et al., 2016). Conversely, overconfidence overestimates an individual’s actual performance (Joo and Durri, 2017). Lack of personal knowledge can easily lead to failure of entrepreneurship, which, in turn, leads to negative emotions (Kinari, 2016). Based on self-regulating attitude theory, specific assessments and desires are functions of unique stimuli that lead to specific emotions and coping responses.

Some studies have found that good service climate may have an impact on employees’ job satisfaction (Schneider et al., 2003), while a general negative affect may result from a poor climate appraisal (Schmit and Allscheid, 1995; Rhodes et al., 2016; Hansen and Steinmetz, 2019). The study assumes that positive entrepreneurial emotion is the emotional reaction to the optimism appraisal processes, while negative entrepreneurial emotion is the emotional reaction to overconfidence. Accordingly, the study tested the following hypotheses:

Hypothesis 2a. Optimism is negatively associated with negative entrepreneurial emotion.

Hypothesis 2b. Optimism is positively associated with positive entrepreneurial emotion.

Hypothesis 3a. Overconfidence is negatively associated with positive entrepreneurial emotion.

Hypothesis 3b. Overconfidence is positively associated with negative entrepreneurial emotion.

### Relationship Between Entrepreneurial Emotion and Entrepreneurship Intention

Although emotional labor is regarded as a predictor of future behavior (Fouquereau et al., 2019), current research on entrepreneurship rarely involves specific behaviors of entrepreneurship (Liu et al., 2019). Recently, some scholars have pointed out that entrepreneurial passion has a significant impact on opportunity recognition and entrepreneurial behaviors (Richards et al., 2019; Wu et al., 2020). Entrepreneurial passion is a part of entrepreneurial emotion, and it may trigger entrepreneurial behavior because it is an individual’s conscious experience, and is conducive to better personal investment in entrepreneurial activities (Li et al., 2017; Obschonka et al., 2019).

In terms of the theory of self-regulating attitude, individual emotional reactions have different effects on behavioral variables. For example, employees’ affective commitment is positively related to service recovery performance, and fear is positively related to idleness (Babakus et al., 2003). In the process of entrepreneurship, positive entrepreneurial emotion can significantly affect the behavior and state of individuals and help entrepreneurs to actively respond to highly uncertain and high-risk entrepreneurial environments, thus triggering risk-seeking behavior. Thus, the individual will form an uplifting mental state, actively engage in entrepreneurship, and will show long-term persistence (Schulte-Holthaus, 2019). On the contrary, individuals who hold negative emotions will show a less optimistic response to the external entrepreneurial environment, hesitate to act, and even miss out on development opportunities (Santos and Cardon, 2019). Hence, this study proposes that positive entrepreneurial emotion and entrepreneurship intention are negatively correlated, while negative entrepreneurial emotion and entrepreneurship intention are positively correlated. Based on the above, the present work argues:

Hypothesis 4. Positive entrepreneurial emotion is negatively associated with entrepreneurship intention.

Hypothesis 5. Negative entrepreneurial emotion is positively associated with entrepreneurship intention.

### The Mediating Role of Emotional Reactions

In existing literature, aside from testing the direct relationship between cognitive bias and entrepreneurship intention, Dali and Harbi (2016) argue that cognitive bias may explain why some individuals’ entrepreneurial behavior results in success while others result in failure. Similarly, some researchers found that enterprise founders tend to have a higher risk bias and perceive lower risk, making strategic decisions faster (Dolarslan et al., 2017). In particular, those studies find that different types of cognitive bias will trigger differences in individual perceptions of the internal and external environment, which will have an impact on their identification and utilization of valuable opportunities. However, they do not explore the mediating role of emotional factors, such as entrepreneurial emotion.

Past studies have demonstrated that positive emotions can be used as additional information to help individuals understand the difficulties optimistically (Huxtable-Thomas et al., 2016; O’Shea et al., 2017). This greatly reduces the risk that enterprise founders perceive in the entrepreneurial process, allowing them to assume that they can control the uncertainties and outcomes of internal and external environments, so that they can make the appropriate entrepreneurial decisions quickly. In contrast to positive emotions, negative emotions reduce the perceived environmental certainty and control of individuals, increase perceived risks, and hinder rapid decision-making behavior (He et al., 2018; Alessa, 2019). Therefore, cognitive bias affects the corresponding entrepreneurial behavior through different types of emotion. Thus, this study argues that the individual with high levels of optimism will react with positive entrepreneurial emotion, thus increasing the likelihood of experiencing entrepreneurship intention. Conversely, the individual with high levels of overconfidence will react with negative entrepreneurial emotion to avoid the threat of entrepreneurship intention.

Based on self-regulating attitude theory, the individual’s cognition and evaluation of the practice of entrepreneurship will lead to the adjustment process of desire-result realization, that is, the individual has achieved the planned results. The individual will produce an emotional reaction, then the individual will produce coping responses in an effort to maintain or raise emotional levels. Restated, positive entrepreneurial emotion and negative entrepreneurial emotion may potentially serve as mediators in the relationship between cognitive bias and entrepreneurship intention in entrepreneurship settings. Given the preceding arguments, the study tested the following hypotheses:

Hypothesis 6. Positive entrepreneurial emotion plays a mediating role in the relationship between optimism and entrepreneurship intention.

Hypothesis 7. Negative entrepreneurial emotion plays a mediating role in the relationship between overconfidence and entrepreneurship intention.

## Research Methods

### Study Samples and Procedure

In this study, a total of 350 questionnaires were distributed to college students who participated in entrepreneurship courses at a Chinese university. A total of 38 invalid questionnaires were excluded, due to incomplete answers and social desirability bias. A total of 312 questionnaires were valid, with an effective questionnaire response rate of 89.14%.

Among the participants, 66.70% were men and 33.32% were women; 33.76% were under 20 years of age, 58.01% were 21–23 years old, and 24 years of age or above accounted for 8.36%; engineering majors accounted for 42.38%, science majors accounted for 8.35%, and social sciences majors accounted for 49.43%. With regard to hukou, 47.42% were urban and 52.6% were rural.

### Measurement

The main scales in this study were adapted from the English literature. After translating English into Chinese, a professional translated it back into English, and then bilinguals compared the original English version with the translated English version, and then the Chinese version for the survey was formed.

#### Optimism

The study adapted 10 items from Scheier et al. (1994) optimism scale to develop a measure of optimism using a seven-point Likert scale (1 = strongly disagree, 7 = strongly agree). In support of construct reliability and validity, the optimism scale has been validated in a prior study for Asian participants, and Cronbach’s alpha was 0.87 (Chang et al., 2019). Sample items include “In uncertain circumstances, I usually expect the best results,” and “I’m always optimistic about the future.”

#### Overconfidence

The study measured overconfidence using Wilson et al.’s (2007) six-item measure of overconfidence using a seven-point Likert scale (1 = strongly disagree, 7 = strongly agree). The scale of overconfidence has been validated in prior research for Chinese participants, and Cronbach’s alpha was 0.86 (Liao and Zheng, 2017). Sample items include “I have the ability to make decisions,” and “I have the ability to solve problems.”

#### Entrepreneurial Emotion

To measure entrepreneurial emotion, the study employed Watson et al.’s (1988) measure of PANAS scales. The PANAS scales have been widely validated in research conducted in China and shows good reliability and validity (Liang and Zhu, 2015; Zhou et al., 2018; Guo et al., 2019). Furthermore, the scale was applied to the study of entrepreneurial emotions in China. The Cronbach’s α values of both positive and negative emotions were >0.6 (He et al., 2017). The study measured positive emotions in six aspects: inspiring, passionate, proud, excited, determined, and active; and measured negative emotions in six aspects: depression, anger, disgust, guilt, nervousness, and fear. A seven-point scale (1 = very weak, 7 = very strong) was used.

#### Entrepreneurship Intention

The study adapted four items from Phan et al.’s (2002) measure of Singapore students’ entrepreneurial propensity to measure entrepreneurship intention using a seven-point Likert scale (1 = strongly disagree, 7 = strongly agree). The scale of Singapore students’ entrepreneurial propensity has been validated in a prior study for Chinese college students, and Cronbach’s alpha was 0.80 (Guo et al., 2013). Sample items include “I will take the initiative to understand the detailed process of starting a business,” and “I’ll start my own business when I get out of school.”

#### Control Variables

Control variables included gender, age, major, and hukou. As previous studies have found that gender and age may affect cognitive bias and entrepreneurial intention (Liao and Zheng, 2017), these variables were added into the model as control variables for empirical analysis.

### Data Analysis Method

First, descriptive statistical analysis, reliability analysis, and correlation analysis of the valid sample were performed through SPSS20. Second, using AMOS 25.0 statistical software, confirmed factor analysis and multi-mediation structure equation modeling were analyzed. Since this study includes multiple mediation models, the bootstrapping method was used in the multi-mediation hypothesis test (Preacher and Hayes, 2008). The study used a bias-corrected method for the confidence interval estimation of total, direct, and indirect effects. When the 95% confidence interval for the indirect effect does not include zero, the mediation effects are considered significant (Selvarajan et al., 2013). Compared with the single-mediation variable model, the advantage of the multi-mediation model is that several mediation variables can be incorporated into the structural equation model at the same time, and the relative effect power of each mediation variable can be discussed simultaneously.

## Results

The descriptive statistics and correlation coefficients of each variable in this study are shown in Table 1.

**TABLE 1:** Descriptives and correlations.

	**Mean**	***SD***	**1**	**2**	**3**	**4**	**5**	**6**	**7**	**8**	**9**
1. Gender	1.33	0.472									
2. Age	1.75	0.597	−0.190**								
3. Major	1.66	0.626	–0.007	0.156**							
4. Hukou	1.96	0.207	–0.032	–0.006	–0.048						
5. OP	4.5535	1.57169	–0.014	–0.032	0.003	0.135*					
6. OC	4.5032	1.68006	–0.020	–0.055	0.039	0.114*	0.015				
7. PEE	4.6667	1.64775	0.000	–0.022	0.059	0.129*	0.230**	−0.121*			
8. NEE	4.3745	1.61832	–0.011	–0.021	0.069	0.151**	0.052	0.236**	0.045		
9. EI	3.9439	1.75752	0.038	–0.095	–0.085	0.023	0.211**	−0.217**	0.193**	−0.250**	

N = 312. OP, Optimism; OC, Overconfidence; PEE, Positive entrepreneurial emotion; NEE, Negative entrepreneurial emotion; EI, Entrepreneurship intention. **p < 0.01, *p < 0.05.

### Reliability and Validity

Confirmatory factor analysis was performed on the following constructs: optimism, overconfidence, positive entrepreneurial emotion, negative entrepreneurial emotion, and entrepreneurship intention. The five-factor model demonstrated a good fit with the data. The various measurement items of constructs were modified from the previous literature. Before the formal survey, the content effect was confirmed by scholars for the questionnaire.

Construct validity includes convergent and discriminant validity. Based on the convergent assessment criteria of Hair et al. (2006), the standardized factor loadings in this study ranged between 0.800 and 0.938, all >0.7; the average variance extracted (AVE) was between 0.785 and 0.870, all >0.5. Moreover, the composite reliability (CR) for each construct was between 0.963 and 0.973, and >0.6. The results show that the scales of this study have convergent validity.

In the assessment criteria for discriminant validity, the mean square root of AVE of each construct should be greater than the correlation coefficient of the constructs, and the number that meets the above criteria must account for 75% of the total (Hair et al., 2006). As shown in Table 2, the average square root of construct AVE was between 0.886 and 0.933, which is greater than the correlation coefficient between the constructs. Therefore, constructs in this study have discriminant validity.

**TABLE 2:** Discriminant validity analysis.

	**AVE**	**OP**	**OC**	**PEE**	**NEE**	**EI**
OP	0.785	**0.886**				
OC	0.855	0.015	**0.925**			
PEE	0.859	0.230**	−0.121*	**0.927**		
NEE	0.812	0.052	0.236**	0.045	**0.901**	
EI	0.870	0.211**	−0.217**	0.193**	−0.250**	**0.933**

N = 312. AVE, Average Variance Extracted. Diagonal elements (bold) are the square roots of AVE. Off-diagonal elements are correlations between constructs. OP, Optimism; OC, Overconfidence; PEE, Positive entrepreneurial emotion; NEE, Negative entrepreneurial emotion; EI, Entrepreneurship intention. **p < 0.01, *p < 0.05.

This study used the Harman single factor method to detect common method variance (CMV) in accordance with the recommendations by Podsakoff et al. (2003). The results found that five factors explained 86.10% of the total variance. The first factor explained 28.91% of the total variance, which did not exceed 50%, so there is no serious CMV in this study.

### Hypothesis Testing

The study tested the overall structural equation model with model fit indexes (Fan et al., 1999; Zhang and Savalei, 2016). The model is ideally fit when χ^2^/d is <3. It is recommended that AGFI and GFI should be above 0.90, NFI and CFI should be >0.9 and SRMR should be <0.08. In addition, RMSEA of <0.08 is acceptable.

The model fit indexes of the overall model in this study are as follows: χ^2^/*d* = 1.293, GFI = 0.902, AGFI = 0.885, CFI = 0.990, PGFI = 0.954, NFI = 0.95, RMSEA = 0.031, and SRMR = 0.095, which is slightly >0.08. Overall, the model fit indexes are above the standard values. It indicates that the model fit the sample data well. Therefore, further testing of the study hypothesis is feasible.

The results of the structural equation model analysis are shown in Table 3. The results show that optimism has a statistically significant positive impact on entrepreneurship intention. Its path β coefficient value is 0.193 (*t* = 3.463, *p* < 0.001); H1a hypothesis is therefore supported. Overconfidence has a statistically significant negative impact on entrepreneurship intention. Its path β coefficient value is −0.148 (*t* = −2.631, *p* < 0.01); H1b hypothesis is therefore supported. However, optimism has no significant effect on negative entrepreneurial emotion. Its path β coefficient value is 0.053 (*t* = 0.922); H2a is therefore not supported. Optimism has a statistically significant positive impact on positive entrepreneurial emotion. Its path β coefficient value is 0.239 (*t* = 4.217, *p* < 0.001); H2b hypothesis is therefore supported.

**TABLE 3:** Path analysis for the research model.

**Path**	**Path coefficients**	**T-value**	**Hypothesis is supported: Yes or No**
H1a: Optimism → Entrepreneurship intention	0.193***	3.463	Yes
H1b: Overconfidence → Entrepreneurship intention	−0.148**	–2.631	Yes
H2a: Optimism → Negative entrepreneurial emotion	0.053	0.922	No
H2b: Optimism → Positive entrepreneurial emotion	0.239***	4.217	Yes
H3a: Overconfidence → Positive entrepreneurial emotion	−0.128*	–2.271	Yes
H3b: Overconfidence → Negative entrepreneurial emotion	0.242***	4.212	Yes
H4: Positive entrepreneurial emotion → Entrepreneurship intention	0.146**	2.603	Yes
H5: Negative entrepreneurial emotion → Entrepreneurship intention	−0.244***	–4.330	Yes

N = 312. ***p < 0.001, **p < 0.01, *p < 0.05.

In addition, overconfidence has a statistically significant negative impact on positive entrepreneurial emotion. Its path β coefficient value is −0.128 (*t* = −2.271, *p* < 0.05); H3a hypothesis is therefore supported. Overconfidence has a statistically significant positive impact on negative entrepreneurial emotion. Its path β coefficient value is 0.242 (*t* = 4.212, *p* < 0.001); H3b hypothesis is therefore supported. Positive entrepreneurial emotion has a statistically significant positive impact on entrepreneurship intention. Its path β coefficient value is 0.146 (*t* = 2.603, *p* < 0.01); H4 hypothesis is therefore supported. Negative entrepreneurial emotion has a statistically significant negative impact on entrepreneurship intention. Its path β coefficient value is −0.244 (*t* = −4.330, *p* < 0.001); H5 hypothesis is therefore supported.

Moreover, to further explore the mediating effect of entrepreneurial emotion between cognitive bias and entrepreneurship intention, the study used the confidence interval method to estimate the confidence interval for indirect, direct, and total effect. The results of the multiple mediating effect test are shown in Table 4.

**TABLE 4:** The multiple mediating effect test.

**Path**			**Bootstrapping**
	**Estimate**	***p*-value**	**Bia-corrected 95% CI**
**Indirect effect**			
H9: Optimism → Positive entrepreneurial emotion n → Entrepreneurship intention	0.035**	0.004	0.010–0.076
H10: Overconfidence → Negative entrepreneurial emotion → Entrepreneurship intention	−0.059***	0.001	−0.113 to −0.026
**Direct effect**			
Optimism → Entrepreneurship intention	0.193***	0.001	0.085–0.295
Overconfidence → Entrepreneurship intention	−0.148*	0.013	−0.260 to −0.034
**Total effect**			
Optimism → Entrepreneurship intention	0.215***	0.001	0.102–0.325
Overconfidence → Entrepreneurship intention	−0.226***	0.001	−0.335 to −0.110

N = 312. Bootstrapping, random sampling 2,000 times. ***p < 0.001, **p < 0.01, *p < 0.05.

For the total effect of optimism on entrepreneurship intention, the lower and upper values of bias-corrected 95% CI are 0.102 and 0.325, respectively. They do not include zero, indicating that the total effect is significantly present. The lower and upper values of bias-corrected 95% CI for direct effect are 0.085 and 0.295, respectively, excluding zero, indicating that the direct effect is significantly present. The lower and upper values of bias-corrected 95% CI for indirect effect are 0.010 and 0.076, respectively, excluding zero, indicating that the indirect effect is significantly present. Therefore, positive entrepreneur emotion has a partial mediation effect between optimism and entrepreneurship intention.

For overconfidence’s total effect on entrepreneurship intention, the lower and upper values of bias-corrected 95% CI are −0.335 and −0.110, respectively, excluding zero, indicating that the total effect is significant. The lower and upper values of bias-corrected 95% CI for direct effect are −0.260 and −0.034, respectively, excluding zero, indicating that the direct effect is significantly present. The lower and upper values of bias-corrected 95% CI for indirect effect are −0.113 and −0.026, respectively, excluding zero, indicating that the indirect effect is significantly present. Therefore, negative entrepreneurial emotion has a partial mediation effect between overconfidence and entrepreneurship intention.

## Discussion

Based on the theory of self-regulating attitude, this study established the conceptual framework of evaluation, emotional reaction, and coping response, and explored the mediation role of entrepreneurial emotion in the relationship between cognitive bias and entrepreneurship intention. This study found that positive entrepreneurial emotion plays a mediating role in the relationship between optimism and entrepreneurship intention. Furthermore, negative entrepreneurial emotion plays a mediating role in the relationship between overconfidence and entrepreneurship intention.

This study found that cognitive factors play an important role in the entrepreneurial process. Entrepreneurship involves a range of behaviors and decision-making processes (Earl, 1996; Shepherd, 2011), and enterprise founders are required to make quick judgments and decisions based on the situation they are facing. Previous studies have shown that founders’ decision-making activities are closely related to their cognitive biases (Sadler-Smith, 2016). Cognitive bias can affect individual decision-making and entrepreneurial behavior. Our study found differences between optimism and overconfidence, and they each have different effects on entrepreneurship intention. This is less explored in past empirical studies, whereas Heger and Papageorge (2018) discuss the impact of optimism and overconfidence on wishful thinking.

This study distinguished the two cognitive biases and found that optimism has a statistically significant positive impact on entrepreneurship intention, which is consistent with Dolarslan et al. (2017). Optimism reflects a positive self-judgment about the ability to control the external environment or predict results. Higher optimism means that individuals have confidence in their abilities, which is critical for entrepreneurs when facing uncertain circumstances. It helps founders face potential dilemmas optimistically and actively drive the entrepreneurial process.

In addition, the study found that overconfidence has a statistically significant negative impact on entrepreneurship intention, which is consistent with Dali and Harbi (2016). The results show that prospective entrepreneurs have a relatively contemptuous view of the difficulties and failures in the entrepreneurial process before engaging in actual entrepreneurial activities. Once the environmental conditions change in the process of entrepreneurship, the entrepreneurship may fail, which in turn may lead to a decrease in entrepreneurship intention. Overconfidence reflects that an individual underestimates the risk of entrepreneurship. Higher overconfidence means that a person perceives less risk. Founders perceive a lower potential risk that will lead to the failure of the venture, thereby reducing the willingness to start a business.

The current study also showed that positive entrepreneurial emotion is an important explanatory factor for entrepreneurial behavior tendencies. These findings are in accordance with past research, which suggested that entrepreneurial traits have an important impact on entrepreneurial intention (Shu et al., 2016). This study introduces entrepreneurial emotion into the framework of the relationship between cognition and behavioral intention and expands the research on emotion in the field of entrepreneurship (O’Shea et al., 2017).

### Theoretical Implications

This study has some theoretical implications. First, this study found the role of cognitive factors in the process of entrepreneurship and revealed that the two cognitive biases of optimism and overconfidence are the key factors that affect entrepreneurial emotion and entrepreneurial intention. In recent years, Chinese governments at all levels continue to promote various initiatives to encourage college students to start their own businesses. Entrepreneurship education has become a compulsory course for many college students. There are endless successful cases of college students’ self-entrepreneurship (Guo et al., 2013). Although there are some differences between college students and social entrepreneurs in demographic characteristics, because college students generally accept entrepreneurship education, and some of them also have practical entrepreneurial experience, it is reasonable to take college students as the research objects of entrepreneurial theory. In the past, college students were also regarded as the research objects of entrepreneurship theory, such as Guo et al. (2013) and Phan et al. (2002). This study used the research logic of the self-regulating process of evaluation, emotional reaction, and coping response and explored how entrepreneurial cognition has an impact on entrepreneurial intention through entrepreneurial emotion. Therefore, the study has expanded the theoretical research on the role of cognition in entrepreneurship.

Second, this study confirmed the applicability of self-regulating attitude theory in the study of entrepreneurship psychology. Existing literature explored the main factors influencing entrepreneurial choices, such as personal, social, and economic factors (Schmitt-Rodermund and Vondracek, 2002; Cubico et al., 2008). However, there is a lack of a theoretical basis for linking these factors to entrepreneurship intention. Based on the theory of self-regulating attitude, these variables are placed in the theoretical framework of self-regulating attitude, and a theoretical model of cognitive bias, entrepreneurial emotion, and entrepreneurship intention was constructed. This empirical study found that cognitive bias leads to individual emotional responses, which include positive entrepreneurial emotion and negative entrepreneurial emotion. Entrepreneurship intention is the result of the behavioral response after the individual’s emotional reaction. This study expands previous research on the relationship between cognitive factors and entrepreneurial behavior and provides a new theoretical basis for the study of entrepreneurship choice.

Moreover, the study focuses on the role of the founder’s emotional characteristics in the entrepreneurial process, revealing the mediating role of entrepreneurial emotion in cognitive bias and entrepreneurship intention. The founder’s emotion in entrepreneurial activities has attracted increasing attention in recent years (O’Shea et al., 2017). Entrepreneurial activities themselves contain a variety of irrational behavior, especially in the development of entrepreneurial decision-making activities and entrepreneurial passion. Existing studies have focused on the role of emotional characteristics in opportunity cognition, entrepreneurial intention, entrepreneurship self-efficacy, etc. (Kumbanaruk, 2008; Breugst et al., 2012), but ignored emotional characteristics’ role in cognitive bias and entrepreneurship intention (Strese et al., 2018). This study empirically analyzed the key role of entrepreneurship emotion in the entrepreneurship intention formation process and contributed to closing the gap of theoretical research on emotional factors in the process of entrepreneurship.

### Practical Implications

The results of this study have the following practical implications. First, in the practice of entrepreneurship management, a correct understanding of cognitive bias should be established. Cognitive bias is a cognitive mechanism that causes individuals to make decisions quickly (Dali and Harbi, 2016). Cognitive bias is a double-edged sword (Dolarslan et al., 2017). As far as positive functions are concerned, it enables entrepreneurs to make decisions without too much time and cognitive resources, even in the face of learning new knowledge in a complex environment. However, its negative function will also result in misjudgment of a situation and in decision-making errors, due to less rational decision-making, cognitive blind spots, and the use of limited information. In turn, it may lead to failure to start a business. Therefore, it is necessary for policymakers to pay attention to improve the entrepreneur’s cognitive ability. In terms of reducing cognitive bias, college students’ entrepreneurs should take practice to identify their own cognitive model, distinguish the difference between optimism and overconfidence, and establish a set of evaluation methods for risk and uncertainty, so as to maintain a positive entrepreneurial emotions and ensure the stimulation and sustainability of entrepreneurial intentions.

Second, in the practice of entrepreneurship management, policymakers should have a clear understanding of entrepreneurial emotion. Positive entrepreneurial emotion is an important entrepreneurial resource, and entrepreneurial emotion may link cognition to entrepreneurial intention and behavior. In the practice of entrepreneurship education and management, organizations and policymakers should have a clear understanding of entrepreneurship emotion, because positive entrepreneurship emotion is an important entrepreneurial resource, and the individual’s intention to start a business is closely related to entrepreneurship emotion. In the future, entrepreneurship education and management practice must strengthen the guidance of the individual’s positive entrepreneurial emotions and help them identify the positive emotions actively, so as to enhance the entrepreneurial intentions and finally promote the actual entrepreneurial behavior.

### Research Limitations and Future Research Recommendations

This paper uses the cross-section research method to collect data. Although the study tested common method bias and the reliability and validity of the related constructs, the causal relationship between these variables is still not completely verified. In the future, researchers should collect longitudinal data through lagging points in time to test the causal relationship among cognitive bias, entrepreneurial emotion, and entrepreneurship intention.

Second, the scales used in this study were mainly from the West, and when these scales are directly referenced in Chinese culture, the scales may not be able to measure the meaning of the concepts. Although the study scales have been back-forward translated, and the study tested their convergence and differentiate validities, follow-up researchers can estimate measurement equivalence of cross-cultural measurement for these scales (Byrne and Watkins, 2003).

Finally, this study’s sample came from college students, and in terms of their attributes, the sample was in line with young people’s entrepreneurship in most countries. However, this study does not investigate actual enterprise entrepreneurs. Because college students and entrepreneurs have different psychological characteristics, it may lead to different perceptions of entrepreneurship and thus result in different conclusions. Follow-up studies can expand the generalizability of this study by conducting surveys of entrepreneurs in real business conditions.

## Data Availability Statement

The datasets generated for this study are available on request to the corresponding author.

## Ethics Statement

Ethical review and approval was not required for the study on human participants in accordance with the local legislation and institutional requirements. The patients/participants provided their written informed consent to participate in this study.

## Author Contributions

Both authors listed have made a substantial, direct and intellectual contribution to the work, and approved it for publication.

## Conflict of Interest

The authors declare that the research was conducted in the absence of any commercial or financial relationships that could be construed as a potential conflict of interest.

## References

[B1] Ajzen I. (1991). The theory of planned behavior. *Organ. Behav. Hum. Decis. Process.* 50 179–211. 10.1016/0749-5978(91)90020-T

[B2] Alessa A. A. (2019). The relationship between education level, gender, emotion and passion on the fear of failure among entrepreneurs. *SMART J. Bus. Manag. Stud.* 15 17–27. 10.5958/2321-2012.2019.00011.3

[B3] Alos-Ferrer C. Garagnani M. Hugelschafer S. (2016). Cognitive reflection, decision biases, and response times. *Front. Psychol.* 7:21. 10.3389/fpsyg.2016.01402 27713710 PMC5031706

[B4] Amarakoon U. Weerawardena J. Verreynne M. L. Teicher J. (2019). Entrepreneurial behaviour: a new perspective on the role of the hr professional. *Pers. Rev.* 48 1809–1829. 10.1108/pr-03-2018-0087

[B5] Babakus E. Yavas U. Karatepe O. Avci T. (2003). The effect of management commitment to service quality on employee affective and performance outcomes. *Official Publ. Acad. Mark. Sci.* 31 272–286. 10.1177/0092070303031003005

[B6] Bagozzi R. P. (1992). The self-regulation of attitudes, intentions, and behavior. *Soc. Psychol. Q.* 55 178–204. 10.2307/2786945

[B7] Bernoster I. Rietveld C. A. Thurik A. R. Torres O. (2018). Overconfidence, optimism and entrepreneurship. *Sustainability* 10:14 10.3390/su10072233

[B8] Bosmans G. Verhees M. De Winter S. (2019). A further validation of the cognitive bias modification effect on trust in middle childhood. *Behav. Ther.* 50 1164–1172. 10.1016/j.beth.2019.04.004 31735250

[B9] Breugst N. Domurath A. Patzelt H. Klaukien A. (2012). Perceptions of entrepreneurial passion and employees’ commitment to entrepreneurial ventures. *Entrepreneursh. Theor. Pract.* 36 171–192. 10.1111/j.1540-6520.2011.00491.x

[B10] Byrne B. M. Watkins D. (2003). The issue of measurement invariance revisited. *J. Cross-Cult. Psychol.* 34 155–175. 10.1177/0022022102250225

[B11] Byrne O. Shepherd D. A. (2015). Different strokes for different folks: entrepreneurial narratives of emotion, cognition, and making sense of business failure. *Entrep. Theory Pract.* 39 375–405. 10.1111/etap.12046

[B12] Cardon M. S. Foo M. D. Shepherd D. Wiklund J. (2012). Exploring the heart: entrepreneurial emotion is a hot topic. *Entrep. Theory Pract.* 36 1–10. 10.1111/j.1540-6520.2011.00501.x

[B13] Chang E. C. Yi S. Liu J. Kamble S. V. Zhang Y. Shi B. (2019). Coping behaviors as predictors of hedonic well-being in asian indians: does being optimistic still make a difference?. *J. Happiness Stud.* 21 289–304. 10.1007/s10902-019-00087-w

[B14] Chaudhary N. (2018). Cross-cultural psychology as a solution to global inequality: optimism, overconfidence, or naivete? A commentary on “the positive role of culture: what cross-cultural psychology has to offer to developmental aid effectiveness research” by symen a. *Brouwers. J. Cross Cult. Psychol.* 49 535–544. 10.1177/0022022117740224

[B15] Chen H. M. Tsai F. S. Ling H. C. (2018). Business area changes and entrepreneurial persistence in ecology- and food-related industries: knowledge heterogeneity and emotion perspectives. *Sustainability* 10:10 10.3390/su10040929

[B16] Cubico S. Bortolani E. Favretto G. (2008). Motivations in entrepreneurial choices: successful and unsuccessful entrepreneurs. *Int. J. Psychol.* 43 782–782. 10.4324/9781315611495-2

[B17] Dali N. Harbi S. (2016). The effect of risk perception and cognitive biases on the evaluation of opportunity in family and non-family entrepreneurs: the case of tunisian entrepreneurs. *J. Enterp. Cult.* 24 281–312. 10.1142/s0218495816500114

[B18] Doern R. Goss D. (2013). From barriers to barring: why emotion matters for entrepreneurial development. *Int. Small Bus. J. Res. Entrep.* 31 496–519. 10.1177/0266242611425555

[B19] Dolarslan E. S. Kocak A. Ozer A. (2017). Bats are blind?” Cognitive biases in risk perception of entrepreneurs. *J. Dev. Entrep.* 22:13 10.1142/s1084946717500212

[B20] Domeier M. Sachse P. (2016). The logic of cognitive biases in the behavioral decision-making process. *Int. J. Psychol.* 51 348–349. 10.4018/978-1-5225-2978-1.ch00326033295

[B21] Earl P. (1996). Paradigms and conventions: uncertainty, decision making and entrepreneurship - choi,yb. *J. Econ. Psychol.* 17 145–148. 10.1016/0167-4870(95)00039-9

[B22] Fan X. Thompson B. Wang L. (1999). Effects of sample size, estimation methods, and model specification on structural equation modeling fit indexes. *Struc. Equ. Model.* 6 56–83. 10.1080/10705519909540119

[B23] Fouquereau E. Morin A. J. S. Lapointe E. Mokounkolo R. Gillet N. (2019). Emotional labour profiles: associations with key predictors and outcomes. *Work Stress* 33 268–294. 10.1080/02678373.2018.1502835

[B24] Giacomin O. Janssen F. Shinnar R. S. (2016). Student entrepreneurial optimism and overconfidence across cultures. *Int. Small Bus. J. Res. Entrep.* 34 925–947. 10.1177/0266242616630356

[B25] Gorgievski M. J. Stephan U. Laguna M. Moriano J. A. (2018). Predicting entrepreneurial career intentions: values and the theory of planned behavior. *J. Career Assess.* 26 457–475. 10.1177/1069072717714541 30443149 PMC6196350

[B26] Grichnik D. Smeja A. Welpe I. (2011). The importance of being emotional: how do emotions affect entrepreneurial opportunity evaluation and exploitation? (vol 76, pg 15, 2010). *J. Econ. Behav. Organ.* 80 680–680. 10.1016/j.jebo.2011.07.001

[B27] Guo Z. Jian’an Z. Jinyun D. (2013). The relationship between entrepreneurial psychological characteristics and entrepreneurial intention of college students: the intermediary effect of coping with employment pressure. *Appl. Psychol.* 19 265–271. 10.13581/006-60202013-03-0265-07

[B28] Guo Z. Xie B. Chen J. Wang F. (2019). The Relationship between opportunities for professional development and counterproductive work behaviors: the mediating role of affective well–being and moderating role of task-contingent conscientiousness. *Int. J. Ment. Health Promot*. 21, 111–122. 10.32604/IJMHP.2019.011040

[B29] Hahn A. M. Simons R. M. Simons J. S. Wiers R. W. Welker L. E. (2019). Can cognitive bias modification simultaneously target two behaviors? Approach bias retraining for alcohol and condom use. *Clin. Psychol. Sci.* 7 1078–1093. 10.1177/2167702619834570 31890350 PMC6936737

[B30] Hair J. Jr. Anderson R. Tatham R. Black W. (2006). *Multivariate Data Analysis*, 6th Edn New York, NY: Macmillan.

[B31] Hansen J. Steinmetz J. (2019). Motivated level of construal: how temperature affects the construal level of state-relevant stimuli. *Motiv. Emot.* 43 434–446. 10.1007/s11031-018-09750-w

[B32] Hayward M. L. A. Forster W. R. Sarasvathy S. D. Fredrickson B. L. (2010). Beyond hubris: how highly confident entrepreneurs rebound to venture again. *J. Bus. Ventur.* 25 569–578. 10.1016/j.jbusvent.2009.03.002

[B33] He L. Yuli Z. Zhenggang S. (2017). A study on the relationship between entrepreneurial sentiment and entrepreneurial behavior tendency. *Res. Dev. Manag.* 29 13–20. 10.13581/j.cnki.rdm.2017.03.002

[B34] He V. F. Siren C. Singh S. Solomon G. von Krogh G. (2018). Keep calm and carry on: emotion regulation in entrepreneurs’ learning from failure. *Entrep. Theory Pract.* 42 605–630. 10.1111/etap.12273

[B35] Heger S. A. Papageorge N. W. (2018). We should totally open a restaurant: how optimism and overconfidence affect beliefs. *J. Econ. Psychol.* 67 177–190. 10.1016/j.joep.2018.06.006

[B36] Hu R. Mao Y. Ye Y. H. (2017). Learning from entrepreneurial failure: emotions, cognitions, and actions. *Int. Entrep. Manag. J.* 13 985–988. 10.1007/s11365-017-0442-y

[B37] Huxtable-Thomas L. A. Hannon P. D. Thomas S. W. (2016). An investigation into the role of emotion in leadership development for entrepreneurs a four interface model. *Int. J. Entrep. Behav. Res.* 22 510–530. 10.1108/ijebr-12-2014-0227

[B38] Joo B. A. Durri K. (2017). Influence of overconfidence, optimism and pessimism on the rationality of the individual investors: an empirical analysis. *Pac. Bus. Rev. Int.* 9 7–13.

[B39] Kinari Y. (2016). Properties of expectation biases: optimism and overconfidence. *J. Behav. Exp. Financ.* 10 32–49. 10.1016/j.jbef.2016.02.003

[B40] Krans J. Bosmans G. Salemink E. De Raedt R. (2019). Cognitive bias modification of expectancies (cbm-e): effects on interpretation bias and autobiographical memory, and relations with social and attachment anxiety. *Cogn. Ther. Res.* 43 1028–1042. 10.1007/s10608-019-10032-z

[B41] Kruglanski A. W. Jasko K. Chernikova M. Milyavsky M. Babush M. Baldner C. (2015). The rocky road from attitudes to behaviors: charting the goal systemic course of actions. *Psychol. Rev.* 122 598–620. 10.1037/a0039541 26192134

[B42] Kumbanaruk T. (2008). The relationships between adversity quotient, emotional quotient, business ethics and stress of small and medium entrepreneurs (smes) in bangkok. *Int. J. Psychol.* 43 538–538. 10.1063/1.5055537

[B43] Lazarus R. S. A. (1991). *Emotion and Adaptation.* New York, NY: Oxford University Press.

[B44] Li J. Chen X. P. Kotha S. Fisher G. (2017). Catching fire and spreading it: a glimpse into displayed entrepreneurial passion in crowdfunding campaigns. *J. Appl. Psychol.* 102 1075–1090. 10.1037/apl0000217 28333500

[B45] Liang Y. Zhu D. (2015). Subjective well-being of Chinese landless peasants in relatively developed regions: measurement using PANAS and SWLS. *Soc. Indic. Res.* 123 817–835. 10.1007/s11205-014-0762-z

[B46] Liao J. Zheng L. (2017). Young heroes: social capital, cognitive biases and entrepreneurial career choice. *J. Scie. Technol. Manag.* 22 31–67.

[B47] Liu X. Y. Wang J. Zhao C. (2019). An examination of the congruence and incongruence between employee actual and customer perceived emotional labor. *Psychol. Mark.* 36 863–874. 10.1002/mar.21241

[B48] Marchetti A. Baglio F. Castelli I. Griffanti L. Nemni R. Rossetto F. (2019). Social decision making in adolescents and young adults: evidence from the ultimatum game and cognitive biases. *Psychol. Rep.* 122 135–154. 10.1177/0033294118755673 29402178

[B49] Neneh B. N. (2019). From entrepreneurial intentions to behavior: the role of anticipated regret and proactive personality. *J. Vocat. Behav.* 112 311–324. 10.1016/j.jvb.2019.04.005

[B50] Obschonka M. Moeller J. Goethner M. (2019). Entrepreneurial passion and personality: the case of academic entrepreneurship. *Front. Psychol.* 9:15. 10.3389/fpsyg.2018.02697 30687165 PMC6335975

[B51] Obschonka M. Silbereisen R. K. Cantner U. Goethner M. (2015). Entrepreneurial self-identity: predictors and effects within the theory of planned behavior framework. *J. Bus. Psychol.* 30 773–794. 10.1007/s10869-014-9385-2

[B52] Obschonka M. Silbereisen R. K. Schmitt-Rodermund E. (2012). Explaining entrepreneurial behavior: dispositional personality traits, growth of personal entrepreneurial resources, and business idea generation. *Career Dev. Q.* 60 178–190. 10.1002/j.2161-0045.2012.00015.x

[B53] Onie S. Gong S. Manwaring E. Grageda D. Webb K. Yuen W. S. (2019). Validation of the australian beverage picture set: a controlled picture set for cognitive bias measurement and modification paradigms. *Aust. J. Psychol.* 36 361–368. 10.1111/ajpy.12272

[B54] O’Shea D. Buckley F. Halbesleben J. (2017). Self-regulation in entrepreneurs: integrating action, cognition, motivation, and emotions. *Organ. Psychol. Rev.* 7 250–278. 10.1177/2041386617705434

[B55] Perez-Lopez M. C. Gonzalez-Lopez M. J. Rodriguez-Ariza L. (2019). Applying the social cognitive model of career self-management to the entrepreneurial career decision: the role of exploratory and coping adaptive behaviours. *J. Vocat. Behav.* 112 255–269. 10.1016/j.jvb.2019.03.005

[B56] Phan P. H. Wong P. K. Wang C. K. (2002). Antecedents to entrepreneurship among university students in singapore: beliefs, attitudes and background. *J. Enterpr. Cult.* 10 151–174. 10.1142/S0218495802000189

[B57] Poblete C. Sena V. de Arroyabe J. C. F. (2019). How do motivational factors influence entrepreneurs’ perception of business opportunities in different stages of entrepreneurship? *Eur. J. Work Organ. Psychol.* 28 179–190. 10.1080/1359432x.2018.1564280

[B58] Podsakoff P. M. MacKenzie S. B. Lee J.-Y. Podsakoff N. P. (2003). Common method biases in behavioral research: a critical review of the literature and recommended remedies. *J. Appl. Psychol.* 88:879. 10.1037/0021-9010.88.5.879 14516251

[B59] Preacher K. Hayes A. (2008). Asymptotic and resampling strategies for assessing and comparing indirect effects in multiple mediator models. *Behav. Res. Methods* 40 879–891. 10.3758/BRM.40.3.879 18697684

[B60] Randerson K. Seaman C. Daspit J. J. Barredy C. (2020). Institutional influences on entrepreneurial behaviours in the family entrepreneurship context: towards an integrative framework. *Int. J. Entrep. Behav. Res.* 26 1–13. 10.1108/ijebr-01-2020-824

[B61] Ravenelle A. J. (2019). We’re not uber:” Control, autonomy, and entrepreneurship in the gig economy. *J. Manage. Psychol.* 34 269–285. 10.1108/jmp-06-2018-0256

[B62] Rhodes R. E. Spence J. C. Berry T. Deshpande S. Faulkner G. Latimer-Cheung A. E. (2016). Understanding action control of parental support behavior for child physical activity. *Health Psychol.* 35 131–140. 10.1037/hea0000233 26214074

[B63] Richards J. Sang K. Marks A. Gill S. (2019). “I’ve found it extremely draining” emotional labour and the lived experience of line managing neurodiversity. *Pers. Rev.* 48 1903–1923. 10.1108/pr-08-2018-0289

[B64] Sadler-Smith E. (2016). The role of intuition in entrepreneurship and business venturing decisions. *Eur. J. Work Organ. Psychol.* 25 212–225. 10.1080/1359432x.2015.1029046

[B65] Santos S. C. Cardon M. S. (2019). What’s love got to do with it? Team entrepreneurial passion and performance in new venture teams. *Entrep. Theory Pract.* 43 475–504. 10.1177/1042258718812185

[B66] Scheier M. F. Carver C. S. Bridges M. W. (1994). Distinguishing optimism from neuroticism (and trait anxiety, self-mastery, and self-esteem): a reevaluation of the life orientation test. *J. Pers. Soc. Psychol.* 67 1063–1078. 10.1037/0022-3514.67.6.1063 7815302

[B67] Schmit M. J. Allscheid S. P. (1995). Employee attitudes and customer satisfaction: making theoretical and empirical connections. *Person. Psychol.* 48 521–536. 10.1111/j.1744-6570.1995.tb01768.x

[B68] Schmitt-Rodermund E. Vondracek F. W. (2002). Occupational dreams, choices and aspirations: adolescents’ entrepreneurial prospects and orientations. *J. Adolesc.* 25 65–78. 10.1006/jado.2001.0449 12009750

[B69] Schneider B. Godfrey E. G. Hayes S. C. Huang M. Lim B.-C. Nishii L. H. (2003). The human side of strategy: employee experiences of strategic alignment in a service organization. *Organ. Dyn.* 32 122–141. 10.1016/S0090-2616(03)00014-7

[B70] Schulte-Holthaus S. (2019). Passion and performance in entrepreneurial contexts: an interest-based approach. *J. Entrep.* 28 201–222. 10.1177/0971355719851895

[B71] Selvarajan T. T. Cloninger P. A. Singh B. (2013). Social support and workBh“family conflict: a test of an indirect effects.model. *J. Vocat. Behav.* 83 486–499. 10.1016/j.jvb.2013.07.004

[B72] Shepherd D. A. (2011). Multilevel entrepreneurship research: opportunities for studying entrepreneurial decision making. *J. Manag.* 37 412–420. 10.1177/0149206310369940

[B73] Shu D. M. Wu H. Z. Feng C. Z. Jiang Y. Z. (2016). Entrepreneurial intention among vocational college students and its relationship with big five traits. *Int. J. Psychol.* 51 912–912. 10.1037/e627722013-357

[B74] Strese S. Keller M. Flatten T. C. Brettel M. (2018). Ceos’ passion for inventing and radical innovations in smes: the moderating effect of shared vision. *J. Small Bus. Manag.* 56 435–452. 10.1111/jsbm.12264

[B75] Trevelyan R. (2008). Optimism, overconfidence and entrepreneurial activity. *Manag. Decis.* 46 986–1001. 10.1108/00251740810890177

[B76] Wang W.-T. Lai W.-Y. Lu C.-T. (2019). Learning from others via team conflicts exploring the impact of individual entrepreneurial characteristics on the construction of entrepreneurial identity. *Int. J. Entrep. Behav. Res.* 10.1108/ijebr-10-2018-0667 [Epub ahead of print].

[B77] Watson D. Clark L. A. Tellegen A. (1988). Development and validation of brief measures of positive and negative affect: the panas scales. *J. Pers. Soc. Psychol.* 54 1063–1070. 10.1037//0022-3514.54.6.1063 3397865

[B78] Wilson F. Kickul J. Marlino D. (2007). Gender, entrepreneurial self–efficacy, and entrepreneurial career intentions: implications for entrepreneurship education. *Entrep. Theory Pract.* 31 387–406. 10.1111/j.1540-6520.2007.00179.x

[B79] Wolfe M. T. Shepherd D. A. (2015). “Bouncing back” from a loss: entrepreneurial orientation, emotions, and failure narratives. *Entrep. Theory Pract.* 39 675–700. 10.1111/etap.12057

[B80] Wu C. Chen Y. C. Meyer M. R. U. (2020). A moderated mediation model of emotional labor and service performance: examining the role of work-family interface and physically active leisure. *Hum. Perform.* 18

[B81] Zacharakis A. L. Shepherd D. A. (2001). The nature of information and overconfidence on venture capitalists’ decision making. *J. Bus. Ventur.* 16 311–332. 10.1016/S0883-9026(99)00052-X

[B82] Zhang X. Savalei V. (2016). Bootstrapping confidence intervals for fit indexes in structural equation modeling. *Struc. Equ. Model.* 23 392–408. 10.1080/10705511.2015.1118692

[B83] Zhou W. Li M. Xin L. Zhu J. (2018). The interactive effect of proactive personality and career exploration on graduating students’ well-being in school-to-work transition. *Int. J. Ment. Health Promot.* 20 41–54. 10.32604/IJMPH.2018.010737

